# Socioeconomic status and incidence of breast cancer by hormone receptor subtype

**DOI:** 10.1186/s40064-015-1282-2

**Published:** 2015-09-17

**Authors:** Tomi F. Akinyemiju, Maria Pisu, John W. Waterbor, Sean F. Altekruse

**Affiliations:** Department of Epidemiology, University of Alabama at Birmingham, Birmingham, AL USA; Division of Preventive Medicine, University of Alabama at Birmingham, Birmingham, AL USA; Comprehensive Cancer Center, University of Alabama at Birmingham, Birmingham, AL USA; Cancer Statistics Branch, Division of Cancer Control and Population Sciences, National Cancer Institute, Bethesda, MD USA

**Keywords:** Breast cancer, Socio-economic status, Disparities, Triple-negative, Cancer

## Abstract

Recent developments in genetics and molecular biology have classified breast cancer into subtypes based on tumor markers of estrogen (ER), progesterone (PR) and human epidermal growth Factor-2 receptors (Her-2), with the basal-like (ER−, PR−, Her2−) subtype commonly referred to as “triple negative” breast cancer (TNBC) being the most aggressive. Prior studies have provided evidence that higher socio-economic status (SES) is associated with increased breast cancer risk, likely due to hormone related risk factors such as parity and hormonal contraceptive use. However, it is unclear if the relationship between SES and overall breast cancer incidence exists within each subtype, and if this association varies by race/ethnicity. Analysis was based on data obtained from the SEER database linked to 2008–2012 American Community Survey data, and restricted to women diagnosed with breast cancer in 2010. The NCI SES census tract SES index based on measures of income, poverty, unemployment, occupational class, education and house value, was examined and categorized into quintiles. Age-adjusted incidence rate ratios were calculated comparing the lowest to the highest SES groups by subtype, separately for each race/ethnic group. We identified 47,586 women with breast cancer diagnosed in 2010. The majority was diagnosed with Her2−/HR+ tumors (73 %), while 12 % had triple negative tumors (TNBC). There was a significant trend of higher incidence with increasing SES for Her2−/HR+ (IRR Highest vs. Lowest SES: 1.32, 95 % CI 1.27–1.39; p value trend: 0.01) and Her2+/HR+ tumors (IRR Highest vs. Lowest SES: 1.46, 95 % CI 1.27–1.68; p value trend: 0.01) among White cases. There was no association between SES and incidence of HR− subtypes (Her2+/HR− or TNBC). Similar associations were observed among Black, Hispanic and Asian or Pacific Islander cases. The positive association between SES and breast cancer incidence is primarily driven by hormone receptor positive tumors. To the extent that neighborhood SES is a proxy for individual SES, future studies are still needed to identify etiologic risk factors for other breast cancer subtypes.

## Background

Whether measured at the individual or residential area level, higher socioeconomic status (SES) has been associated with higher breast cancer incidence (Pudrovska and Anikputa 2012; Krieger et al. 2010; Vainshtein 2008; Yost et al. 2001), with the most consistent results found among White women in the U.S. (Yu et al. 2014; Palmer et al. 2012; Borugian et al. 2011; Clegg et al. 2009; Reynolds et al. 2005). This association may reflect differences in exposure to breast cancer risk factors. For instance, women of higher SES in general have lower parity, greater use of exogenous hormones, and greater alcohol consumption, all established risk factors for breast cancer (Palmer et al. 2012; Suzuki et al. 2005; Heck and Pamuk 1997). Most of these studies have classified breast cancer as a single disease, although recent genetic and molecular analyses have established the existence of several subtypes of breast cancer, based on ER, PR, and Her status. In order of increasingly aggressive behavior and worse prognosis, the subtypes are: Luminal A (ER+ and/or PR+, Her2−), Luminal B (ER+ and/or PR+, Her2+), and basal-like (ER−, PR−, Her2−), this last subtype also commonly referred to as “triple negative” breast cancer or TNBC (Morris and Mitchell 2008; Amend et al. 2006).

The prevalence of TNBC and HR− tumors has been shown to be higher among pre-menopausal African-American women, and it is associated with more aggressive disease and shorter survival (Stark et al. 2010; Fregene and Newman 2005; Agboola et al. 2013; Gukas et al. 2008; Carey et al. 2006). Since African-American women are more likely to belong to low SES groups, it is important to determine if SES differences explain the higher prevalence of HR− tumor subtypes in this racial group. Such studies could inform etiologic studies that consider each subtype separately to identify risk factors or biological mechanisms that can be addressed as part of intervention studies to reduce the prevalence of aggressive subtypes, and thus reduce racial disparities in breast cancer outcomes. However, only a few studies have examined the association between SES and breast cancer subtypes (Parise et al. 2009; Banegas et al. 2014; Sineshaw et al. 2014), and most of those studies utilized data from a single US state (Parise et al. 2009; Banegas et al. 2014). Furthermore, characterization of SES has been inconsistent, with SES measured at different geographic levels and different definitions of SES used.

The current study examines the association between SES and breast cancer subtypes among the major US racial groups, using an expanded population-based dataset with a validated composite census tract-level SES index. Our research evaluates whether the positive association between SES and breast cancer incidence exists in all breast cancer subtypes, and within each racial group, utilizing a valid measure of SES and the Surveillance, Epidemiology and End Results (SEER) database.

## Methods

### Data source

The data for this analysis was obtained from the National Cancer Institute SEER database linked to the 2008–2012 American Community Survey data. The SEER 18 population-based dataset includes all breast cancer cases diagnosed in 2010 in the following SEER cancer registries: Atlanta, Connecticut, Detroit, Hawaii, Iowa, New Mexico, San-Francisco-Oakland, Seattle-Puget Sound, Utah, Los Angeles, San Jose-Monterey, rural Georgia, Greater California, Kentucky and New Jersey. About 28 % of the U.S. population is covered by SEER, although the regions included tend to be more urban and suburban compared with the general U.S. population.

### Individual level data

The main outcome variable for this analysis is incidence of first primary breast cancer among women ages 20 years and older. The subtype classification of breast cancer is based on SEER variables relating to the hormone receptor status of tumors recorded by the SEER program. TNBC is defined as ER−/PR−/Her2−; Luminal A is defined as ER+ and/or PR+ HER2−; and Luminal B is defined as ER+ and/or PR+, Her2+. Details of the variable coding for ER, PR and Her2 have been published elsewhere (Howlader et al. 2014), and are available through the SEER website (http://seer.cancer.gov/seerstat/databases/ssf/). The Her2 recode variable was available only as of 2010; and our analysis was based on data from that year. Other individual level variables assessed include age at diagnosis, and race/ethnicity (NH-White: non-Hispanic White, NH-Black: non-Hispanic Black, NH-API: non-Hispanic Asian or Pacific Islander, and Hispanic). This study was considered exempt by the Institutional Review Board of the University of Alabama at Birmingham, since analysis was based on publicly available, non-identifiable SEER data.

### Census tract SES

The NCI registry-based census tract SES (NCI-SES) index was used in this analysis as our SES measure. Detailed methodology regarding this index has been published previously. Factor analyses was performed on SES- related measures identified by Yost et al. (2001), with higher scores corresponding to higher SES. The index is based on measures of income, poverty, unemployment, occupational class, education and house value. The SES scores obtained from factor analysis were divided into quintiles with roughly equal proportions of the population in each category, ranging from lowest SES to highest SES. The SES classification of each census tract was assigned to all cancer cases residing in that census tract at the time of diagnosis.

### Statistical analysis

Age-adjusted incidence rates (and their standard errors) per 100,000 women were calculated for breast cancer cases using SEER*Stat (Version 8.1.5). Incidence rate ratios and 95 % confidence intervals were calculated using the Tiwari method (Tiwari et al. 2006) and age-adjusted to the U.S. standard population stratified by race/ethnicity (Tiwari et al. 2006). Frequencies and percentages were calculated for each age group, race/ethnicity, SES, disease stage and grade overall and by subtype.

## Results

In 2010, 47,586 women were diagnosed with breast cancer and met our eligibility criteria; the majority of cases (n = 34,753, 73 %) were diagnosed with Her2−/HR+ tumors, and 5764 (12 %) had triple negative tumors (TNBC). Table 1 provides descriptive statistics for the breast cancer subtypes. NH-Blacks comprised about 9 % of all breast cancer cases, and of those, 22 % were diagnosed with the TNBC subtype and 61 % with Her2+/HR− subtype. In contrast, NH-Whites comprised 72 % of all breast cancer cases, with 11 % diagnosed with TNBC subtype and 76 % with Her2−/HR+ subtype. About 14 % of API cases and 13 % of Hispanic breast cancer cases were of TNBC subtypes. About 17 % of all cases were diagnosed at stages III and IV, however a higher proportion of TNBC cases (21.3 %) and Her2+/HR− cases (29.6 %) were diagnosed at the later stages. Overall 25 % of all cases resided in the highest SES census tracts, compared with 14 % in the lowest SES census tracts.

**Table 1:** Socio-Demographic and tumor characteristics of breast cancer cases, SEER 2010

Study variables	Total = 47,586 N (%)	Her2+/HR+ N = 4891 (10.3)	Her2+/HR− N = 2178 (4.6)	Her2−/HR+ N = 34,753 (73.0)	TNBC N = 5764 (12.1)
Age (years)					
<50	10,093 (21.2)	1444 (14.3)	563 (5.6)	6574 (65.1)	1512 (14.9)
50–64	18,081 (38.0)	1937 (10.7)	968 (5.4)	12,836 (70.9)	2340 (12.9)
65–74	10,412 (21.9)	860 (8.3)	354 (3.4)	8127 (78.1)	1071 (10.3)
≥75	9000 (18.9)	650 (7.2)	293 (3.3)	7216 (80.2)	841 (9.3)
Race/ethnicity					
NH-White	34,228 (71.9)	3334 (9.7)	1357 (4.0)	25,867 (75.6)	3670 (10.8)
NH-Black	4503 (9.5)	504 (11.2)	271 (6.0)	2748 (61.0)	980 (21.8)
NH-API	185 (0.4)	27 (15.0)	10 (5.4)	123 (66.5)	25 (13.5)
Hispanic	8670 (18.2)	1026 (11.8)	540 (6.2)	6015 (69.4)	1089 (12.6)
AJCC Stage					
I	23,635 (49.7)	1976 (8.4)	727 (3.1)	18,862 (79.8)	2070 (8.8)
II	14,992 (31.5)	1674 (11.2)	727 (3.1)	10,274 (68.5)	2316 (15.5)
III	5539 (11.6)	750 (13.5)	441 (7.9)	3466 (62.6)	882 (15.9)
IV	2332 (4.9)	344 (14.8)	205 (8.8)	1434 (61.5)	349 (14.9)
Unknown	1067 (2.2)	145 (13.6)	72 (6.7)	716 (67.1)	134 (12.6)
Grade					
Low	10,562 (22.2)	388 (3.7)	27 (0.3)	10,010 (94.8)	137 (1.3)
Medium	19,841 (41.7)	1907 (9.6)	491 (2.5)	16,470 (83.0)	973 (4.9)
High	15,055 (31.6)	2336 (15.5)	1521 (10.1)	6784 (45.1)	4414 (29.3)
Unknown	2128 (4.5)	260 (12.2)	139 (6.5)	1489 (69.9)	240 (11.3)
NCI census tract SES					
Group 1—low	6575 (13.8)	667 (10.1)	350 (5.3)	4533 (68.9)	1025 (15.6)
Group 2	8254 (17.4)	853 (10.3)	403 (4.8)	5880 (71.2)	1118 (13.5)
Group 3	9520 (20.0)	1010 (10.6)	438 (4.6)	6968 (73.2)	1104 (11.6)
Group 4	10,583 (22.2)	1085 (10.3)	460 (4.4)	7837 (74.1)	1201 (11.4)
Group 5—high	12,006 (25.2)	1214 (10.1)	502 (4.2)	9053 (75.4)	1237 (10.3)

**Fig. 1:**
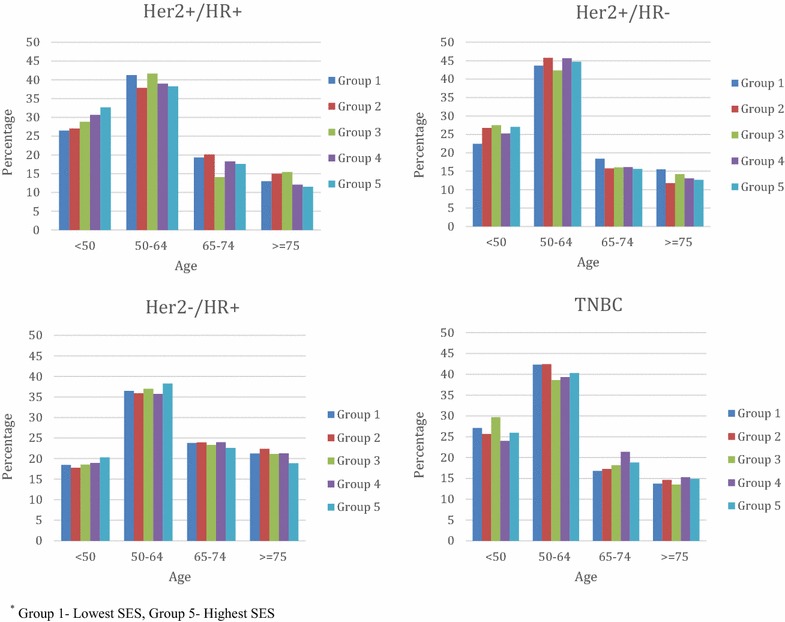
Distribution of breast cancer subtypes by age and SES, SEER 2010. *Group 1* lowest SES, *Group 5* highest SES

Figure 1 depicts the distribution of breast cancer cases within each subtype stratified by age group and SES. The majority of cases occurred between ages 50–64 years regardless of subtype, although there was a higher proportion of younger cases (<50 years) and lower proportion of older cases (≥75 years) in the Her2+/HR+ subtype, and the inverse (lower proportion of younger cases and higher proportion of older cases) in the Her2−/HR+ subtype. In general, there were no substantial increases in the proportion of breast cancer cases with increasing SES, except among younger cases with Her2+/HR+ and a smaller increase among younger cases with Her2−/HR+ subtype.

In Table 2, age-adjusted incidence rates (IR) and incidence rate ratios (IRR) are presented for each subtype by census-tract SES, stratified by race/ethnicity. Overall, there was an increased risk of breast cancer with increasing SES in each racial group (p value trend <0.05), with estimates ranging from 21 % among NH-Blacks (IRR Group 5 vs. 1:1.21, 95 % CI 1.07–1.35), 37 % among NH-Whites (IRR Group 5 vs. 1: 1.37, 95 % CI 1.22–1.55), 33 % among NH-API (IRR Group 5 vs. 1: 1.33, 95 % CI 1.19–1.49), and 37 % among Hispanic cases (IRR Group 5 vs. 1: 1.37, 95 % CI 1.24–1.52). When stratified by subtype, the increased risk associated with SES appeared to be driven by associations in the HR+ subtypes; there was no significant increased risk in the Her2+/HR− or TNBC subtype for any racial group. In contrast, there was significantly increased risk associated with increasing SES among NH-Blacks (IRR Group 5 vs. 1:1.36, 95 % CI 1.17–1.57), NH-Whites (IRR Group 5 vs. 1: 1.32, 95 % CI 1.27–1.39), NH-API (IRR Group 5 vs. 1: 1.46, 95 % CI 1.27–1.68) and Hispanic (IRR Group 5 vs. 1: 1.52, 95 % CI 1.35–1.72) cases with Her2−/HR+ subtype. Only NH-White (IRR Group 5 vs. 1: 1.46, 95 % CI 1.27–1.68), NH-API (IRR Group 3 vs. 1: 1.49, 95 % CI 1.05–2.16) and Hispanic (IRR Group 4 vs. 1: 1.41, 95 % CI 1.07–1.86) cases with Her2+/HR+ subtypes showed an increased risk with higher SES.

**Table 2:** Breast cancer incidence rate (IR) and incidence rate ratios (IRR) by hormone receptor subtype and SES, SEER 2010

Socio-economic status	IR^a^	Breast cancer subtype IRR				
		Total	Her2+/HR+	Her2+/HR−	Her2−/HR+	TNBC
NH-Black						
Group 1—low (Ref)	134.8	–	–	–	–	–
Group 2	141.0	1.05 (0.96–1.13)	1.12 (0.87–1.44)	0.77 (0.54–1.08)	1.06 (0.95–1.17)	1.08 (0.91–1.28)
Group 3	153.2	1.14 (1.04–1.24)^¶^	1.17 (0.89–1.53)	0.85 (0.58–1.22)	1.22 (1.08–1.36)^¶^	0.99 (0.82–1.21)
Group 4	148.2	1.09 (0.99–1.21)	1.21 (0.89–1.62)	0.98 (0.65–1.45)	1.07 (0.94–1.22)	1.16 (0.94–1.43)
Group 5—high	162.5	1.21 (1.07–1.35*)* ^¶^	1.14 (0.79–1.61)	0.95 (0.56–1.53)	1.36 (1.17–1.57*)* ^¶^	0.88 (0.67–1.16)
*P* *value trend*		*0.025*	*0.16*	*0.82*	*0.11*	*0.76*
NH-White						
Group 1—low (Ref)	137.2	–	–	–	–	–
Group 2	144.2	1.05 (1.00–1.10*)* ^¶^	1.17 (1.00–1.36)	1.10 (0.87–1.39)	1.05 (0.99–1.11)	0.95 (0.83–1.08)
Group 3	153.0	1.12 (1.06–1.17)^¶^	1.27 (1.09–1.49)^¶^	1.11 (0.89–1.39)	1.13 (1.07–1.19)^¶^	0.92 (0.81–1.05)
Group 4	164.2	1.19 (1.15–1.25)^¶^	1.32 (1.14–1.52)^¶^	1.17 (0.94–1.45)	1.22 (1.16–1.28)^¶^	0.98 (0.87–1.12)
Group 5—high	177.0	1.37 (1.22–1.55)^¶^	1.46 (1.27–1.68)^¶^	1.23 (0.99–1.52)	1.32 (1.27–1.39)^¶^	0.98 (0.87–1.11)
*P* *value trend*		*0.007*	*0.001*	*0.004*	*0.009*	*0.96*
NH-API						
Group 1—low (Ref)	93.1	–	–	–	–	–
Group 2	103.2	1.11 (0.98–1.26)	1.24 (0.85–1.84)	1.03 (0.62–1.72)	1.14 (0.97–1.33)	0.85 (0.56–1.28)
Group 3	116.3	1.24 (1.11–1.41)^¶^	1.49 (1.05–2.16)^¶^	1.46 (0.94–2.32)	1.25 (1.08–1.46)^¶^	0.84 (0.57–1.26)
Group 4	122.2	1.31 (1.16–1.48)^¶^	1.12 (0.78–1.63)	1.09 (0.98–1.77)	1.41 (1.21–1.63)^¶^	1.07 (0.74–1.57)
Group 5—high	127.6	1.33 (1.19–1.49)^¶^	1.32 (0.94–1.91)	1.03 (0.65–1.68)	1.46 (1.27–1.68)^¶^	1.12 (0.78–1.62)
*P* *value trend*		*0.002*	*0.44*	*0.85*	*0.001*	*0.31*
Hispanic						
Group 1—low (Ref)	91.2	–	–	–	–	–
Group 2	96.6	1.06 (0.97–1.15)	1.11 (0.86–1.43)	1.18 (0.83–1.67)	1.06 (0.95–1.18)	0.98 (0.79–1.22)
Group 3	111.5	1.22 (1.12–1.33)^¶^	1.27 (0.98–1.65)	0.85 (0.57–1.29)	1.27 (1.14–1.41)^¶^	1.16 (0.93–1.44)
Group 4	115.1	1.26 (1.15–1.39)^¶^	1.41 (1.07–1.86)^¶^	0.97 (0.63–1.47)	1.38 (1.23–1.54)^¶^	0.79 (0.60–1.04)
Group 5—high	125.2	1.37 (1.24–1.52)^¶^	0.97 (0.69–1.35)	1.16 (0.75–1.79)	1.52 (1.35–1.72)^¶^	1.09 (0.83–1.43)
*P* *value trend*		*0.002*	0.74	0.79	0.001	0.97

Italicized *P*-value are from Chi-square tests for linear trend for each subtype by SES

^¶^ *P* value <0.05, indicates that the rate ratio is significantly different than the rate for Group 1

^a^Incidence rates are per 100,000 and age-adjusted to the 2000 US standard population

## Discussion

This study examines the association between census tract level SES and the incidence of breast cancer subtypes among different racial groups in the U.S. Consistent with other published studies, we observed higher rates of breast cancer incidence among women residing in higher SES areas compared with lower SES areas (Vainshtein 2008; Yost et al. 2001; Krieger et al. 2006) for HR+ subtypes (Her2−/HR+ and Her2+/HR+), but not HR− subtypes. This association was observed consistently among all racial/ethnic groups. Our findings suggest that distinct etiologic pathway(s) exist that contributing to the risk of HR− compared with HR+ breast cancer subtypes. Differential distribution of such exposure(s) may be the source of higher prevalence of HR− subtypes among younger, NH-Black, and low SES women (Howlader et al. 2014; Clarke et al. 2012; Amirikia et al. 2011; Kurian et al. 2010; Lund et al. 2010).

The association between SES and breast cancer incidence overall has been well characterized. Women of higher SES may experience higher breast cancer incidence due to the higher circulatory hormones as a result of unique reproductive patterns such as earlier menarche, lower parity and later age at first birth (La Vecchia et al. 1993; Kelsey et al. 1993; Ewertz and Duffy 1988). Higher SES women are also more likely to obtain routine mammography screening due to better access to preventive healthcare, thereby increasing the detection of breast cancer in this group. Although a few recent studies have examined the distribution of breast cancer subtypes (Parise et al. 2009; Banegas et al. 2014; Sineshaw et al. 2014), only recently have scientists begun to examine traditional breast cancer risk factors in relation to breast cancer hormonal subtypes. For instance, recent studies have reported that the association between parity (Aktipis et al. 2014; Ritte et al. 2013; Ambrosone et al. 2014; Phipps et al. 2011) and breastfeeding (Ambrosone et al. 2014; Palmer et al. 2014; Redondo et al. 2012), and breast cancer likely varies by hormone receptor status. Higher parity was associated with increased risk of TNBC, and longer duration of breastfeeding was associated with reduced risk of TNBC (Ritte et al. 2013; Redondo et al. 2012). Women of low SES may be at higher risk for TNBC because they tend to have higher parity (Mosher et al. 2012; Stanford and Smith 2013; Burr and Bean 1996) but lower breastfeeding rates (Kitsantas et al. 2011; Flacking et al. 2010; Flacking et al. 2007). These are patterns that have been observed among Non-Hispanic black women relative to other racial/ethnic groups (Hamilton et al. 2014; Martin et al. 2010; Belanoff et al. 2012; Singh et al. 2007).

Although there were no associations observed between SES and HR− breast cancer subtypes in this study, future studies may contribute to our understanding of this phenomenon by examining individual level SES in relation to breast cancer subtypes. While census-tract SES has been validated as a concrete measure of neighborhood SES, it may not fully capture individual level variation in SES. Other areas of investigation may also focus on population differences in the prevalence of both reproductive, lifestyle and environmental risk factors that could increase the risk of HR− breast cancer. For instance, poor diet and lack of physical activity have been shown to be highly associated with the risk of metabolic syndrome, a cluster of metabolic abnormalities including obesity, insulin resistance, dyslipidemia and hypertension (Martinez et al. 2014; Clark et al. 2013; Cubbin et al. 2001). Metabolic syndrome has been associated with TNBC in several studies (Vargas-Hernandez et al. 2013; Davis and Kaklamani 2012; Maiti et al. 2010), and is hypothesized to increase risk of TNBC through the association with leptin and adiponectin levels that disrupt cell signaling pathways involved in cell cycle regulation, angiogenesis and cell proliferation (Davis and Kaklamani 2012).

If confirmed by studies adequately powered to account for confounders, these are highly modifiable risk factors that could be targeted by primary prevention programs. In addition, there may be distinct breast tumorigenic pathways driven at least partially by an underlying genetic or epigenetic pathway, giving rise to HR− tumors.

This is the first population-based study to characterize the association between SES and breast cancer subtypes by racial groups in the U.S. The large sample size allowed for stratification by hormone receptor subtype in assessing the SES-subtype association, and the use of SEER data ensured that all variables were standardized. Our results show that HR− breast cancer incidence is independent of SES and highlights the need for further studies to fully characterize this relationship and identify etiologic factors. Studies are needed to examine individual level SES and associated risk factors (e.g. obesity) for HR− tumors, and multilevel studies should examine both individual and neighborhood SES. Longitudinal studies can be done to account for the induction period between exposure and breast cancer development. There are several limitations of this study. First, there was no data on individual level SES, highlighting the need for further studies to determine if the association between census-tract SES and HR− breast cancer is partially or fully mediated by individual SES. Second, we lacked information on individual risk factors for breast cancer. This is a well-known limitation of cancer registry data studies, and we hope that future studies can overcome this limitation through the use of primary datasets or by using registry data linked with individual level risk factor data.

## Conclusion

Contrary to the well-known association of higher breast cancer incidence with higher SES, we found that the incidence rate of HR− breast cancer subtypes did not increase with increasing SES, as measured by the census-tract level data. There are several other etiologic pathways that may lead to HR− breast cancer incidence, and future studies are needed to fully characterize those to better inform breast cancer prevention strategies.
